# Determinants for utilization and transitions of long-term care in adults 65+ in Germany: results from the longitudinal KORA-Age study

**DOI:** 10.1186/s12877-018-0860-x

**Published:** 2018-07-31

**Authors:** Kathrin Steinbeisser, Eva Grill, Rolf Holle, Annette Peters, Hildegard Seidl

**Affiliations:** 1 Institute of Health Economics and Health Care Management, Research Center for Environmental Health, Helmholtz Zentrum München, Ingolstädter Landstr., Neuherberg, 85764 Germany; 2 Institute for Medical Informatics, Biometry and Epidemiology, Ludwig-Maximilians-Universität München, Marchioninistr. 17, 81477 Munich, Germany; 3 Institute of Epidemiology II, Helmholtz Zentrum München, German Research Center for Environmental Health, Ingolstädter Landstr. 1, 85764 Neuherberg, Germany

**Keywords:** Long-term care, Types of care, Health care utilization, Determinants, Transition, Longitudinal analysis, Generalized estimating equations

## Abstract

**Background:**

Societies around the world face the burden of an aging population with a high prevalence of chronic conditions. Thus, the demand for different types of long-term care will increase and change over time. The purpose of this exploratory study was to identify determinants for utilization and transitions of long-term care in adults older than 65 years by using Andersen’s Behavioral Model of Health Services Use.

**Methods:**

The study examined individuals older than 65 years between 2011/2012 (t_1_) and 2016 (t_2_) from the population-based Cooperative Health Research in the Region of Augsburg (KORA)-Age study from Southern Germany. Analyzed determinants consisted of predisposing (age, sex, education), enabling (living arrangement, income) and need (multimorbidity, disability) factors. Generalized estimating equation logistic models were used to identify determinants for utilization and types of long-term care. A logistic regression model examined determinants for transitions to long-term care over four years through a longitudinal analysis.

**Results:**

We analyzed 810 individuals with a mean age of 78.4 years and 24.4% receiving long-term care at t_1_. The predisposing factors higher age and female sex, as well as the need factors higher multimorbidity and higher disability score, were determinants for both utilization and transitions of long-term care. Living alone, higher income and a higher disability score had a significant influence on the utilization of formal versus informal long-term care.

**Conclusion:**

Our results emphasize that both utilization and transitions of long-term care are influenced by a complex construct of predisposing, enabling and need factors. This knowledge is important to identify at-risk populations and helps policy-makers to anticipate future needs for long-term care.

**Trial registration:**

Not applicable

**Electronic supplementary material:**

The online version of this article (10.1186/s12877-018-0860-x) contains supplementary material, which is available to authorized users.

## Background

In Europe, the number of people in need of long-term care (LTC) will increase dramatically by 2060 [1]. This trend is caused by the demographic change, which leads to an aging population and is expected to increase the prevalence of disability and chronic conditions [2].

To prepare the health care system for this urgent public health problem, it is important to understand the reasons for utilization and transitions of LTC over time. LTC can be defined as assistance with daily activities for people who are not fully capable of self-care on a long-term basis [3]. Daily activities consist of activities of daily living (ADL), such as bathing or grooming, and instrumental activities of daily living (IADL), such as shopping or doing housework [4]. LTC can be informal or formal. Informal LTC is defined as assistance from family members, friends or neighbors, whereas formal LTC encompasses institutional and home-based LTC provided by a skilled nurse or institution, as well as paid services for household support [3].

In 2015, about 2.86 million people in Germany were in need of LTC. Of those, 72.6% received home-based LTC, whereas 27.4% received institutional LTC [5]. To provide support for LTC services, Germany’s statutory nursing care insurance was introduced in 1995. People who apply for support are evaluated based on the amount of assistance they need for ADLs and IADLs. This is determined by a needs assessment, conducted by the statutory Health Insurance Medical Service (MDK) [6, 7]. Based on the minimum time of assistance needed in minutes per day, one of the three care levels (I, II, III) is assigned to the person applying for support, according to legal guidelines from 2012 to 2016 [8]. Further information on Germany’s nursing care insurance can be found in Additional file 1.

To detect determinants for the utilization of health care services, such as those received from physicians or hospitals, Andersen’s Behavioral Model of Health Services Use (ABMHS) [9] has often been employed. ABMHS is also applicable for identifying determinants for the utilization of LTC [10]. The model distinguishes predisposing, enabling and need factors. Predisposing factors represent demographic and social characteristics of individuals, such as age or education. Enabling factors consist of factors such as income or living arrangement and can either support or impede utilization of LTC. For example, living with a family member, a potential informal caregiver, can lead to the utilization of informal LTC. Need factors are defined as people’s physical and psychological health and functional status. If they deteriorate, they can support utilization of LTC [9, 10]. To date, research has shown that older people’s utilization of LTC is associated with different factors, such as higher age [11–14], female sex [11, 14, 15] and impairments in daily activities due to chronic conditions [11] or disability [12–14, 16, 17]. Further differences could be found in the utilization of types of LTC. Older adults with higher income [13, 18] and who live alone [11–14, 17, 19] are more likely to receive formal than informal LTC. Studies have mainly focused on determinants for the utilization of institutional LTC, because this type of LTC causes high costs and thus is highly relevant for policy-makers to consider [20, 21]. Another focus of research has been people with specific diseases, such as Parkinson disease [22]. Less is known about the utilization of LTC by community-dwelling older adults without specific diseases and thus should to be investigated.

To prepare the health care system for future demands of LTC services, factors that determine the transitions of LTC (i. e. changes from no or one type of LTC to another) are even more important to identify. Therefore, longitudinal studies are necessary. To date, little is known about factors that determine transitions of LTC on an individual basis [23].

The main objectives of this study are to identify relevant determinants for (1) utilization and (2) transitions of LTC over a time period of four years in adults older than 65 years in Germany. This approach allows a direct comparison of factors that are associated with current utilization of LTC and factors that might determine a transition to LTC over time. Furthermore, we would like to investigate the average amount of LTC received by individuals and the changes over four years. The results of this study may help to identify at-risk populations and plan future demands for LTC services.

## Methods

### Study design and participants

The present Cooperative Health Research in the Region of Augsburg (KORA)-Age study was based on data from KORA research, a platform for population-based surveys and follow-up studies of health care research in Germany [24]. The KORA-Age study is a follow-up of all participants born before 1944 from four independent cross-sectional samples, performed between 1984 and 2001. Participants were randomly selected from population registries of persons living in the Bavarian city Augsburg along with two adjacent counties (population in 2012: 639,000). The participants’ random selection was ensured through taking random numbers representing people from population registration. The original four independent cross-sectional samples, which serve as a basis for the KORA-Age study, were drawn in a two-stage procedure where first the city of Augsburg and 16 communities within its adjacent communities were selected by cluster-sampling. After that, stratified random sampling was performed within each community of the adjacent counties. Therefore, four cross-sectional health surveys comprised independent random samples [24, 25]. The KORA-Age study was approved by the Ethics Committee of the Bavarian Medical Association. Written informed consent was obtained from all participants and all investigations were conducted according to the principles expressed in the Declaration of Helsinki. Details on data collection, study design and sampling method are explained elsewhere [24, 26, 27].

**Fig. 1:**
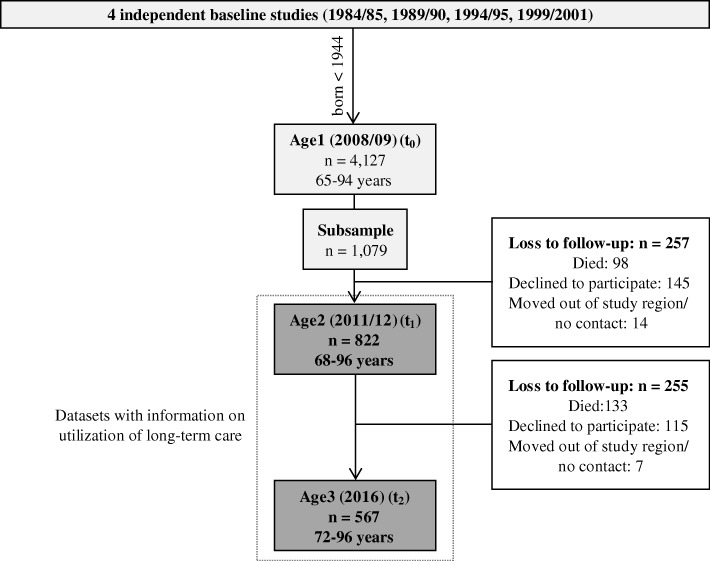
Flow chart about KORA-Age population

In 2008 (Age1/ t_0_), a sample of 4127 persons participated in a standardized computer-assisted telephone interview with detailed questions on morbidity and sociodemographic information. If the participant was unable to answer the questions, a proxy participant was interviewed. Out of the 4127 participants, a gender- and age-stratified subsample of 1079 individuals with 100 persons per stratum was invited for physical examinations and further follow-ups. Between 2011/2012 (Age2/ t_1_), 822 people (response rate 76.2%) received a further telephone-interview and physical examinations. Due to drop-outs, 567 individuals were followed-up in 2016 (Age3/ t_2_) (see Fig. 1). Since information on utilization of LTC was collected only in t_1_ and t_2_, we considered these follow-up studies for analyses. For the telephone interview’s quality assurance, pilot studies of the survey questions were conducted, interviewers were trained and certified and interviews were recorded. To correct implausible values, all interviews of t_1_ and one third of t_2_ were audited again.

### Measures: utilization and transitions of long-term care

Utilization of LTC was measured by asking the respondents if they received LTC due to their health status within the last three months from (1) a home nursing service (i. e. assistance with ADLs), (2) paid services for household support (i. e. assistance with IADLs) (3) family members, friends or neighbors. Receiving LTC was defined as receiving (1), (2), (3) or a combination of them. Receiving formal LTC was defined as either receiving (1), (2) or both. Informal LTC was equivalent to (3). If individuals received both formal and informal LTC, they were considered as receiving formal LTC [23]. Individuals living in skilled nursing facilities were considered as receiving formal LTC, since they received mainly formal assistance with ADL and IADL [3]. A transition to LTC was defined as the change from no LTC at t_1_ to receiving LTC (informal or formal) at t_2_. The amount of formal and informal LTC was calculated in minutes per day, based on respondent estimates.

### Measures: determinants for utilization and transitions of long-term care

All possible determinants for utilization and transitions of LTC were assessed during the telephone-interview. They were identified through literature research and classified as predisposing (age, sex, education), enabling (living arrangement, income) and need (multimorbidity, disability) factors according to ABMHS [9, 28]. Age referred to age at telephone interview. Information on education was obtained from the four baseline KORA-samples and categorized as “low” (≤ 9 years), “middle” (10–11 years) and “high” (≥ 12 years). Living arrangement was dichotomized as living “alone” and “not alone”. Individuals living in skilled nursing facilities were referred to as living not alone, since formal caregivers were available day and night. We calculated income with monthly tax-deducted household-based income at an individual level [29]. In order to allow for non-linear effects, income was grouped in quartiles: “< 875€”, “875 - 1,124€”, “1,125 - 1,374€”, “≥ 1375€”. Data on 13 chronic conditions were collected according to the self-report generated Charlson Comorbidity Index [30] (heart, joint, lung, gastrointestinal, kidney, liver disease, Diabetes Mellitus, stroke, cancer, HIV) and three additional questions on hypertension, neurological and eye diseases [31]. Multimorbidity was defined as the number of chronic conditions, ranging from 0 to 13. Disability scores were assigned using the Stanford Health Assessment Questionnaire Disability Index (HAQ-DI), which assesses impairments in ADLs and IADLs [32–34]. The instrument consists of 20 questions in eight domains (dressing and grooming, standing up, eating, walking, hygiene, reach, grip, and activities). Responses range between 0 (“no difficulty”) and 3 (“unable to perform”). Each domain’s score was built from the highest score in the current domain. The HAQ-DI was computed by calculating the mean of all eight domains and was reported as a continuous value ranging from 0.000 to 3.000.

### Statistical analysis

Subject characteristics at t_1_, dropouts between t_1_ and t_2_, average amount of LTC and transitions of LTC were analyzed descriptively. To examine both differences in individuals with and without LTC and between non-dropouts and dropouts, subject characteristics were compared using Pearson’s chi-square tests for independence for categorical variables and t-tests for continuous variables.

To investigate which determinants were associated with the utilization of LTC at t_1_ or at t_2_ (cross-sectional analysis with repeated measurements), we used a two-stage generalized estimating equation (GEE) logistic model with an unstructured working correlation matrix. GEE accounts for repeated measurements and their accompanying intra-subject correlation [35]. It calculates population-averaged effects. Contrary, mixed models calculate subject-specific effects [36]. Stage 1 analyzed utilization versus no utilization of LTC (informal or formal). For the analysis, observations from t_1_ and t_2_ were summed up. For t_1_, values of the seven independent variables (age, sex, education, living arrangement, income, multimorbidity, disability score) at time point t_1_ were used; for t_2_, values of time-varying variables (age, living arrangement, disability score) at t_2_ and values of fixed variables (sex, education, income, multimorbidity) of t_1_ were used. Due to limited data access, multimorbidity was used of t_1_. Since income for people older than 65 years in Germany mainly is based on retirement pension [37], it varies only on a small scale. Thus, income was considered as a fixed variable. Stage 2 analyzed utilization of formal LTC versus utilization of informal LTC. Only individuals that received LTC were considered for Stage 2 to examine the determinants of formal versus informal LTC.

To investigate which determinants were associated with transitions of LTC (longitudinal analysis), we conducted a two-stage logistic regression model [38]. To enable a clear outcome, the groups with a transition from LTC to no LTC or LTC to LTC were excluded from the logistic regression model. Stage 1 assessed determinants for transition from no LTC at t_1_ to LTC (informal or formal) at t_2_. Stage 2 examined the difference in receiving formal versus informal LTC. For Stage 2, only individuals who had a transition from no LTC at t_1_ to LTC at t_2_ could be considered. In order to predict the likelihood of transition to LTC at t_2_, both logistic regression models were conducted using the seven independent variables from t_1_. All independent variables were tested for multicollinearity in all models. Odds ratios (OR) and 95% confidence intervals (CI) were calculated. Variables were considered significant at *p*-value ≤0.05, equal for all analyses in this study.

Missing values in the dependent variable “utilization of LTC” in both informal and formal LTC at t_1_ (*n* = 12) and at t_2_ (*n* = 1) reduced the final sample size for the cross-sectional analysis from 822 to 810 and for the longitudinal analysis to 563 individuals (complete case analysis) [39, 40]. For the cross-sectional and longitudinal analyses, a total of 53 missings in independent variables at t_1_ (multimorbidity (*n* = 9/810, 1.1%), disability score (*n* = 1/810, 0.1%), income (*n* = 43/810, 5.3%)) and one at t_2_ (disability score (*n* = 1/563, 0.2%)) were identified. Single stochastic regression imputation using predictive mean matching was conducted through fully conditional specification method [41]. The imputation model assumes that missing values are missing at random, meaning that they are conditionally independent from the unobserved value and underlie an arbitrary missing data pattern [42, 43]. Imputation was based on information available from the variables sex, age, education, living arrangement and utilization of LTC. All statistical analyses were performed using SAS software, release 9.3 (SAS Institute, Cary, NC).

## Results

### Characteristics of study sample

Table 1 shows the characteristics of the total sample and groups by utilization of LTC at t_1_. From 810 individuals, 402 (49.6%) were female. Mean age was 78.4 years, ranging from 68 to 94 years. Individuals receiving LTC (*n* = 197, 24.4%) were more likely to be older (mean age: 82.5 vs. 77.1 years), female (65.7% vs. 44.4%), live alone (50.5% vs. 29.9%), have more chronic conditions (3.2 vs. 2.3) and a higher disability score (1.219 vs. 0.274). Out of 197 people who received LTC, 152 (77.2%) had no care level, 32 (16.2%) care level I, ten (5.1%) care level II and three (1.5%) care level III.

**Table 1:** Characteristics of participants at t_1_ stratified by utilization of long-term care (*n* = 810)

		N	Total	No LTC	LTC	*P*-value
			(*n* = 810)	(75.6%)	(24.4%)	
Predisposing factors						
Age in years	total	810	78.4 (6.4)	77.1 (6.0)	82.5 (6.0)	**< 0.0001** ^a^
Sex	female	810	402 (49.6%)	272 (44.4%)	130 (65.7%)	**< 0.0001** ^b^
Education	low	810	548 (67.7%)	415 (67.8%)	133 (67.2%)	0.9416^b^
	middle		153 (18.9%)	114 (18.6%)	39 (19.7%)	
	high		109 (13.5%)	83 (13.6%)	26 (13.1%)	
Enabling factors						
Living arrangement	alone	810	283 (34.9%)	183 (29.9%)	100 (50.5%)	**< 0.0001** ^b^
Per capita income in €/ month	total	767	1138.4 (579.0)	1138.9 (564.1)	1137.0 (624.4)	0.9691^a^
	< 875 €		181 (23.6%)	134 (23.1%)	47 (25.0%)	0.9505^b^
	875–1124 €		199 (26.0%)	150 (25.9%)	49 (26.1%)	
	1125–1374 €		188 (24.5%)	144 (24.9%)	44 (23.4%)	
	≥ 1375 €		199 (26.0%)	151 (26.1%)	48 (25.5%)	
Need factors						
Multimorbidity in no. of chronic conditions	total	801	2.5 (1.5)	2.3 (1.4)	3.2 (1.6)	**< 0.0001** ^a^
Disability score (HAQ-DI)	total	809	0.504 (0.7)	0.274 (0.4)	1.219 (0.9)	**< 0.0001** ^a^

*LTC* long-term care, *HAQ-DI* Health Assessment Questionnaire Disability Index

Bold numbers: significant at *p* ≤ 0.05

Data presented as n (%)/ mean (standard deviation) | any discrepancies in percentages due to rounding | ^a^ based on t-test ^b^ based on chi2-test

### Dropout analysis

At t_2_, 246 (30.4%) of those who responded at t_1_ dropped out. One-hundred-twenty-nine had died, 110 had declined to participate and seven had moved out of the study region or contact was not possible. Of the dropouts, 40.0% (*n* = 99) had already received LTC at t_1_; of non-dropouts 17.6% (*n* = 99). Except for sex, all characteristics of individuals were significantly different between dropouts and non-dropouts. Dropouts were more likely to be older, live alone and have a higher multimorbidity and higher disability score (see Additional file 2).

### Determinants for utilization of long-term care

Table 2 reports determinants for utilization of LTC versus no utilization of all 1373 observations at t_1_ (*n* = 810) and t_2_ (*n* = 563) (Stage 1). If individuals received LTC, we compared formal versus informal LTC (Stage 2). Of the 378 observations with LTC, 228 (60.3%) reported receiving informal LTC, while 150 (39.7%) reported receiving formal LTC. Regarding Stage 1, the predisposing factors higher age (OR: 1.09, CI: 1.05–1.13), female sex (OR: 1.91, CI: 1.25–2.91) and high education as compared with low education (OR: 2.18, CI: 1.23–3.84) were significantly associated with the utilization of LTC. Among the enabling factors, adults living alone had higher odds (OR: 1.71, CI: 1.14–2.55) to receive LTC, whereas income was not significantly associated with LTC. The need factors multimorbidity (OR: 1.21, CI: 1.07–1.36) and disability score (OR: 8.72, CI: 6.23–12.20) had also a significant influence on the utilization of LTC.

**Table 2:** Influence of ABMHS factors on utilization of long-term care – GEE logistic model (1373 observations)

		Stage 1: LTC vs. no LTC^a^			Stage 2: formal vs. informal LTC^b^		
		Odds ratio	95% confidence interval	*P*-value	Odds ratio	95% confidence interval	*P*-value
Predisposing factors							
Age in years		1.09	[1.05; 1.13]	**< 0.0001**	1.02	[0.98; 1.07]	0.2904
Sex (ref: male)	female	1.91	[1.25; 2.91]	**0.0027**	1.12	[0.66; 1.90]	0.6690
Education (ref: low)	middle	1.23	[0.76; 2.01]	0.4009	0.83	[0.44; 1.54]	0.5540
	high	2.18	[1.23; 3.84]	**0.0074**	1.82	[0.85; 3.91]	0.1251
Enabling factors							
Living arrangement (ref: not alone)	alone	1.71	[1.14; 2.55]	**0.0097**	1.71	[1.02; 2.85]	**0.0418**
Per capita income/ month (ref: < 875 €)	875–1124 €	1.05	[0.62; 1.79]	0.8459	2.17	[1.09; 4.34]	**0.0282**
	1125–1374 €	0.68	[0.40; 1.14]	0.1426	2.94	[1.42; 6.08]	**0.0037**
	≥ 1375 €	0.82	[0.47; 1.43]	0.4832	2.84	[1.33; 6.07]	**0.0071**
Need factors							
Multimorbidity in no. of chronic conditions		1.21	[1.07; 1.36]	**0.0026**	0.90	[0.77; 1.05]	0.1881
Disability score (HAQ-DI)		8.72	[6.23; 12.20]	**< 0.0001**	2.45	[1.80; 3.33]	**< 0.0001**

ABMHS: Andersen’s Behavioral Model of Health Services Use (predisposing, enabling, need factors) | GEE: generalized estimating equation | LTC: long-term care | HAQ-DI: Health Assessment Questionnaire Disability Index

Bold numbers: significant at p ≤ 0.05

Sample for generalized estimating equation: sum of t_1_- (*n* = 810) and t_2_-sample (*n* = 563)

^a^Stage 1: Determinants for utilization of long-term care

Model includes all observations of t_1_ and t_2_ (*n* = 1373) to examine determinants for utilization of long-term care (independently of type of long-term care); observations are grouped by utilization of either long-term care (*n* = 378) or no long-term care (*n* = 995)

^b^Stage 2: Determinants for utilization of formal vs. informal long-term care

Model includes all observations with utilization of long-term care (*n* = 378) to examine the determinants for utilization of formal vs. informal long-term care; observations are grouped by utilization of either formal (*n* = 150) or informal long-term care (*n* = 228)

Regarding Stage 2, living alone (OR: 1.71, CI: 1.02–2.85) increased the odds for utilization of formal LTC. Whereas income showed no significant association with the utilization of LTC in general, an income higher than 874 Euros increased the odds for utilization of formal LTC. Additionally, a higher disability score (OR: 2.45, CI: 1.80–3.33) was strongly related to the utilization of formal LTC.

### Amount of long-term care

Table 3 presents the average amount of LTC in minutes per day received by individuals who participated at both t_1_ and t_2_ (*n* = 563). Overall, the number of individuals receiving LTC increased from t_1_ to t_2_. At both t_1_ and t_2_, more individuals received informal than formal LTC. Between t_1_ and t_2_, assistance with ADL increased more than four times from 20.9 (standard deviation (SD): 15.8) to 89.9 (SD: 231.6) minutes per day.

**Table 3:** Average amount of long-term care per day at t_1_ and t_2_ of long-term care users

	t_1_ (*n* = 563)			t_2_ (*n* = 563)		
	N	Minutes	SD	N	Minutes	SD
Home based long-term care						
Informal long-term care	77	65.4	(117.0)	152	105.3	(202.7)
Formal long-term care	33	52.2	(164.5)	60	68.7	(183.2)
of that ADL	12	20.9	(15.8)	35	89.9	(231.6)
of that IADL	25	58.9	(189.2)	37	26.4	(58.6)
Skilled nursing facility^a^	4			8		

ADL: activities of daily living | IADL: instrumental activities of daily living | SD: standard deviation

Multiple answers for informal and formal long-term care (IADL, ADL) were possible

^a^Amount of long-term care for skilled nursing facilities was not assessed in questionnaires

### Determinants for transitions of long-term care

**Fig. 2:**
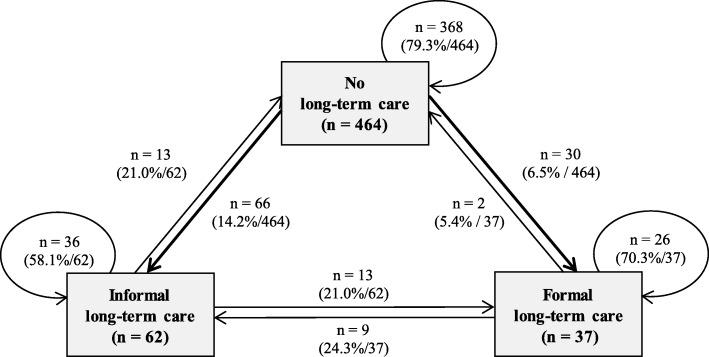
Transitions of long-term care from t_1_ to t_2_ by type of care

Transitions of LTC are displayed in Fig. 2. Out of the 563 individuals who participated at both t_1_ and t_2_, 122 (21.7%) had a transition from one status to another, whereas 441 (78.3%) remained in the same status. Of the 464 persons with no LTC at t_1_, 66 (14.2%) had a transition to informal LTC and 30 (6.5%) a transition to formal LTC. Individuals remaining with LTC at both t_1_ and t_2_ (*n* = 85, 15.1%), as well as those having a transition from LTC to no LTC (*n* = 15, 2.7%), were excluded from the longitudinal analysis.

Table 4 reports determinants for transition from no LTC at t_1_ to LTC at t_2_ (Stage 1). If there was a transition, determinants for utilization of formal versus informal LTC were analyzed (Stage 2). Regarding Stage 1, the predisposing factors higher age (OR: 1.15, CI: 1.09–1.21) and female sex (OR: 2.14, CI: 1.16–3.92) were significantly associated with a transition to LTC, whereas education had no significant influence. Among the enabling factors, living arrangement had no influence on a transition to LTC. However, individuals with an income higher than 1374 Euro per month had lower odds for a transition to LTC than those with an income lower than 875 Euro (OR: 0.32, CI: 0.14–0.78). The need factors multimorbidity (OR: 1.32, CI: 1.09–1.60) and disability score (OR: 5.82; CI: 2.83–11.95) were also significantly associated with a transition to LTC.

**Table 4:** Influence of ABMHS factors on transition to long-term care – logistic regression model (*n* = 464)

		Stage 1: LTC vs. no LTC^a^			Stage 2: formal vs. informal LTC^b^		
		Odds ratio	95% confidence interval	*P*-value	Odds ratio	95% confidence interval	*P*-value
Predisposing factors							
Age in years		1.15	[1.09; 1.21]	**< 0.0001**	1.11	[1.01; 1.23]	**0.0350**
Sex (ref: male)	female	2.14	[1.16; 3.92]	**0.0143**	2.51	[0.84; 7.53]	0.1000
Education (ref: low)	middle	1.34	[0.67; 2.70]	0.4131	0.68	[0.21; 2.25]	0.5291
	high	1.50	[0.59; 3.77]	0.3941	0.44	[0.07; 2.91]	0.3970
Enabling factors							
Living arrangement (ref: not alone)	alone	1.41	[0.75; 2.68]	0.2876	0.67	[0.22; 1.97]	0.4616
Per capita income/ month (ref: < 875 €)	875–1124 €	1.06	[0.52; 2.17]	0.8776	1.17	[0.36; 3.81]	0.7997
	1125–1374 €	0.60	[0.27; 1.30]	0.1905	1.24	[0.34; 4.54]	0.7415
	≥ 1375 €	0.32	[0.14; 0.78]	**0.0117**	1.38	[0.27; 7.15]	0.6989
Need factors							
Multimorbidity in no of chronic conditions		1.32	[1.09; 1.60]	**0.0045**	1.11	[0.80; 1.55]	0.5300
Disability score (HAQ-DI)		5.82	[2.83; 11.95]	**< 0.0001**	0.69	[0.24; 1.99]	0.4959

LTC: long-term care | HAQ-DI: Health Assessment Questionnaire Disability Index

Bold numbers: significant at *p* ≤ 0.05

^a^Stage 1: Determinants for transition to long-term care for individuals with no long-term care at t_1_

Model includes all individuals with a transition from no long-term care (t_1_) to either informal or formal long-term care (t_2_) (*n* = 96) in comparison to individuals without a transition (*n* = 368) to examine the determinants for a transition from no long-term care to any type of long-term care

^b^Stage 2: Determinants for utilization of formal vs. informal long-term care

Model includes all individuals with a transition from no long-term care (t_1_) to long-term care (t_2_) (*n* = 96) to examine the determinants for the utilization of formal versus informal long-term care; individuals are grouped by utilization of either formal (*n* = 30) or informal long-term care (*n* = 66) at t_2_

Regarding Stage 2, the odds for the utilization of formal versus informal LTC increased with each year of rising age (OR. 1.11; CI: 1.01–1.23). Other variables in this analysis did not show significant associations with a transition to LTC.

## Discussion

This study investigated the effects of predisposing, enabling and need factors as determinants for utilization and transitions of LTC in a population-based sample. To the knowledge of the authors, this is the first study to examine determinants for utilization, as well as for transitions of LTC, in older adults. The predisposing factors higher age and female sex, as well as the need factors higher multimorbidity and higher disability score, were determinants for both utilization and transitions of LTC. Living alone, higher income and higher disability score had a significant influence on the utilization of formal versus informal LTC. Overall, our findings are in line with other international studies that have identified determinants for utilization [11–14] or transitions of LTC [23, 44, 45].

Regarding the utilization of LTC, we found that the predisposing factor higher age was an important determinant. A number of studies have found that utilization of LTC increases with higher age [11–14], due to the higher care needs of this group [11]. Our results showed that female sex also increased the probability to receive LTC, which is confirmed by previous research [11, 14, 15]. This phenomenon might be related to females’ higher support-seeking attitude in health care services (e. g. physician visits, hospital stays), which has been identified to be independent of females’ health status [46]. In our study, individuals with high education were more likely to receive LTC than those with low education. This is consistent with findings on utilization of health care services that show that individuals with higher education might be more aware of existing supports [47, 48]. However, research on utilization of LTC shows that higher education is either associated with higher [12, 13] or lower utilization of LTC [49], or that it changes over time [50]. One possible explanation for these inconsistencies is that education is often completed during young adulthood, years before people reach old age, when LTC is normally received. The study’s results indicated that living alone increased the probability to receive LTC. The enabling factor living arrangement is reportedly a significant determinant for utilization of LTC [11, 12, 19, 51]. Much evidence shows that individuals living alone receive more frequently formal than informal LTC [11–14, 17, 19]. Availability of a person in the same household may reduce the demand for formal LTC [12, 13, 17]. We indicated that higher income was associated with the utilization of formal LTC, which is consistent with previous findings that higher income facilitates using paid LTC services [13, 18].

Among the need factors, our results revealed that higher multimorbidity had a considerable impact on the utilization of LTC. Van den Bussche et al. [52] have examined 46 chronic diseases and state that the need of LTC for adults older than 65 years in Germany increases with every disease. However, they have focused on the need of LTC, defined by having a care level. Our study emphasized that multimorbidity has an impact on the utilization of LTC in a study sample that includes also individuals who did not fulfil the prerequisites for receiving a care level. In a study which has examined the association of 23 chronic diseases with the utilization of LTC, more than 90% of the diseases had a significant influence on the outcome [11]. These findings emphasize the importance of considering chronic diseases, especially multimorbidity, as determinants for utilization of LTC. An interesting result was that with a higher disability score, the odds of utilization of any LTC, as well as of formal LTC, increased dramatically. These findings are consistent with previous research, which equally has defined disability with impairments in ADLs and IADLs [12–14, 16, 17]. According to literature, impairments in ADLs are one of the major determinants for utilization of LTC, especially for formal LTC [16].

Regarding the average amount of LTC, our study showed that individuals receive more informal than formal LTC. Considering a similar definition of informal and formal LTC as our study, Wimo et al. [53] and Katz et al. [19] accord with our observations. Comparing the amount of LTC of our study with the amount of the two studies in minutes per day, it is notable that both other studies report higher values. This could be caused due to our healthier study sample, whereas Wimo et al. have focused on older people with dementia and Katz et al. on older people with disability. None of the studies have analyzed a change of the amount of LTC over time. In our study we could show that over a period of four years the amount of LTC increased. An explanation might be the higher disability with higher age, which increases the demand for LTC [11–13] and can be seen in Additional file 3.

Regarding the determinants for a transition to LTC, our findings provide further evidence about determinants for transition from no LTC to LTC. Most studies in this field have mainly focused on determinants for transitions only to [54, 55] and from [56] skilled nursing facilities, and for individuals with specific diseases or restrictions, such as dementia [57, 58] or palliative patients [59, 60]. In contrast, determinants for transitions from no LTC to LTC, independent of the type of LTC, have been rarely investigated in a community-dwelling population [23, 44, 61]. We found consistency in the determinants of utilization and transitions of LTC. Determinants for both were higher age and female sex, as well as higher multimorbidity and disability score. Higher age was a determinant for a transition from no LTC to LTC, as well as for the utilization of formal versus informal LTC. Geerlings et al. [23] have analyzed determinants for transitions from no LTC to informal LTC and from no or informal LTC to formal LTC. In agreement with our results, they have found that higher age is a determinant for both transitions from no LTC to informal or formal LTC.

Interestingly, a study on transitions of LTC in twelve European countries (including Germany) which controlled for determinants similar to those we controlled, has shown that higher age is a significant determinant for transition from no or informal to formal LTC [44]. These findings show that higher age has to be considered on country-level as a strong determinant for transition to LTC. We also identified female sex as a determinant for transition to LTC. This could be explained by evidence that women seek more support in health care services [46]. Contrary to Pan et al. [45], who have shown that income had no significant association with a transition to LTC, our findings state that having a higher income decreased the transition from no LTC to LTC. These results must be interpreted with caution. One possible explanation could be that income is not as important as wealth in retirement age [45]. Because data on wealth was unavailable, this information could not be considered in the current study. It is notable that all need factors had a significant influence on transition to LTC, which is also shown by Geerlings et al. [23]. Over a period of four-and-a-half years, Koller et al. [62] have revealed that older adults in Germany with more than three chronic diseases had higher odds of transition to “need of LTC”, defined as receiving a care level. Despite analyzing the need, instead of utilization of LTC, this finding is consistent with our study. The influence of multimorbidity could already be shown in our study’s sample, which also included individuals who did not fulfil the prerequisites for receiving a care level. An international study [61] shows that multimorbidity has an influence on transition from no or informal LTC to formal LTC in eight European countries, which confirms our results on a macro level and emphasizes the high importance to consider multimorbidity as a determinant for transition to LTC. Regarding disability, research indicates that this need factor is associated with transition to LTC [23], especially to formal LTC [63]. Impairments in ADL and IADL hinder self-care and likely decrease independence in daily life.

### Strengths and limitations

Analyzing both outcomes utilization and transitions of LTC within the same study sample allowed us to show the relationships between determinants for utilization and transitions of LTC. As part of the KORA-studies, instruments were carefully chosen and standardized assessments were conducted. The GEE logistic model allowed to consider the longitudinal approach of this study and could clearly identify determinants for utilization of LTC. To date, this methodology could rarely be used to identify determinants for utilization of LTC, due to limitations of cross-sectional data, as presented in previous studies.

Some limitations of the present study have to be acknowledged. First, information based on self-reports and might be susceptible to information bias. However, previous studies have shown that self-reports are a valid method to collect data on utilization of health care services [64].

In considering the generalizability of this study, it is essential to mention that our study sample was selected to explore the determinants for utilization and transitions of LTC in older adults. The oversampling of men and older adults thus allowed us to examine influential factors in a relatively large sample of older community-dwelling adults, not those of the general population. Although the city of Augsburg and its two surrounding counties are not representative for whole Germany, we could show that our determinants for utilization of LTC are similar to previous findings with larger sample sizes [11, 12].

Furthermore, our study had dropouts due to death and refusals. The stratified analysis by dropout could show that dropouts had a higher utilization of LTC and a poorer health status at t_1_. As a result, the strength of the associations between determinants for utilization and transitions of LTC is likely to have been underestimated.

It has also to be acknowledged that this study did not identify significant determinants for transitions of LTC within formal versus informal LTC apart from age. This might have been due to the small sample size for this sub-analysis and has to be examined in future studies. Nevertheless, the majority of determinants of formal and informal LTC show similar trends as in previous findings [23, 45].

Also, the GEE logistic model could only estimate population-, rather than subject-specific correlations [35, 36]. To examine determinants for utilization of LTC on an individual or class basis, other models (e. g. mixed models) should be used in future studies.

It would also be interesting to look at other patterns, such as the transition from informal to formal LTC. These patterns should be analyzed in future studies with bigger sample sizes.

Due to limited data access, it was not possible to include information on multimorbidity at t_2_ and on different stages of the included chronic diseases. This would have made our analysis more accurate [52]. Research states that chronic diseases deteriorate over time [62], which could lead to systematic underestimation of multimorbidity as a determinant for utilization of LTC. Future studies should examine transitions to LTC in larger samples over a longer time period, including stages rather than only the number of chronic diseases.

## Conclusions

In conclusion, our results emphasize that both utilization and transitions of LTC are influenced by a complex construct of predisposing, enabling and need factors. Identified at-risk populations should receive more attention, especially women, adults with higher age and a poorer health status. The increasing demand of LTC services in society highlights the existing public health problem and therefore the importance of efforts toward mindful planning for future needs in this sector.

## Additional files

Additional file 1: Germany’s nursing care insurance. (DOCX 23 kb)

Additional file 2: Characteristics of participants stratified by dropout. (DOCX 19 kb)

Additional file 3: Time-varying characteristics of participants. (DOCX 18 kb)

## Abbreviations

ABMHS: Andersen’s Behavioral Model of Health Services Use
ADL: Activities of daily living
CI: 95% confidence interval
FCS: Fully conditional specification
GEE: Generalized estimating equations
HAQ-DI: Health Assessment Questionnaire Disability Index
IADL: Instrumental activities of daily living
KORA: Cooperative Health Research in the Region of Augsburg
LTC: Long-term care
MDK: Health Insurance Medical Service
OR: Odds ratio
SD: Standard deviation
