# Supplementary material for: Determinants for utilization and transitions of long-term care in adults 65+ in Germany: results from the longitudinal KORA-Age study
Source: BMC Geriatr. 2018 Jul 31;18:172. doi: 10.1186/s12877-018-0860-x (PMC6069853; doi:10.1186/s12877-018-0860-x)
Supplement: Supplementary file 1 — Germany’s nursing care insurance. (DOCX 23 kb) [file 12877_2018_860_MOESM1_ESM.docx]

### **Additional file 1**: Germany’s nursing care insurance

Germany’s nursing care insurance covers both home-based and institutional long-term care (LTC) services [1]. The German LTC Insurance Act of 1994 states that home-based LTC should be used rather than institutional LTC [1–3]. To encourage informal caregivers to provide LTC to their relatives or friends, nursing care insurance gives financial support for home-based informal LTC (“Pflegegeld”). Furthermore, assistance from formal caregivers (“Pflegesachleistung”) or a combination of financial support and formal LTC for home-based LTC can be provided. If home-based LTC is not possible, financial support for institutional LTC is provided [1].

Up to 2016, three care levels (I, II, III) existed. Since 2017, five care grades have replaced the former care levels [2]. As this study based on care levels rather than care grades, we describe the situation as it was between 2011/2012 and 2016, the years of data collection. For the assignment to one of the care levels, the requirements are categorized into need for assistance with activities of daily living (ADL), such as bathing or grooming, and instrumental activities of daily living (IADL), such as shopping or doing housework [4]. In Table A1, the minimum time of assistance needed in minutes per day for ADL and IADL for each care level and the amount of financial support for home-based and institutional LTC are displayed for the year 2015/2016 [1]. The prerequisites for receiving a care level were similar in 2011/2012 and 2016, except that requirements in 2016 considered more cognitive impairments. The benefits increased only minimally between 2011/2012 and 2016. Until a person is assigned to a care level, it is not possible to file claims for support from nursing care insurance. As need for LTC changes over time, the care level can be re-evaluated at a later time point [1, 3].

Table A1: Assignment of care levels by minimum time of needed assistance per day

| **Care level** | **Minimum time of needed assistance in minutes/ day** | **Benefits in Euro/ month for^a^** | | | | |
| --- | --- | --- | --- | --- | --- | --- |
|  |  | **Home-based long-term care** | | | **Institutional long-term care** | |
|  |  | Pflegegeld^b^ | Pflegesachleistung^c^ |  | |  |
| I | 90  (of that 45 Min for ADL) | 244 | 468 | 1064 | |  |
| II | 180  (of that 120 Min for ADL) | 458 | 1144 | 1330 | |  |
| III | 300  (of that 240 Min for ADL) | 728 | 1612 | 1612 | |  |
| III+ | 300  additional support at night | / | 1995 | 1995 | |  |

ADL: activities of daily living

^a^ all values are based on the minimum of benefits/ month in 2016, according to Sozialgesetzbuch §§36-37,43 [1]

^b^ The term “Pflegegeld” is defined as financial support from nursing care insurance for a person who receives

home-based informal long-term care, provided by family or friends

^c^ The term “Pflegesachleistung” is defined as financial support from nursing care insurance for a person who

receives home-based formal long-term care; it is directly paid to the professional nursing care service

**References**

1. Bundesministerium der Justiz und für Verbraucherschutz. Sozialgesetzbuch XI. Soziale Pflegeversicherung. Stand Februar 2016: SGB XI; 2016.

2. Naegele G. 20 Jahre Verabschiedung der Gesetzlichen Pflegeversicherung: Eine Bewertung aus sozialpolitischer Sicht. Bonn: Friedrich-Ebert-Stiftung; 2014.

3. Marburger H. Die Pflegeversicherung. 6th ed. Stuttgart: Richard Boorberg Verlag; 2016.

4. MDK. Information on needs assessment. 2014. https://www.mdk-bayern.de/mdk-international/english-en.html. Accessed 10 Jul 2017.
