# Supplementary material for: Determinants for utilization and transitions of long-term care in adults 65+ in Germany: results from the longitudinal KORA-Age study
Source: BMC Geriatr. 2018 Jul 31;18:172. doi: 10.1186/s12877-018-0860-x (PMC6069853; doi:10.1186/s12877-018-0860-x)
Supplement: Supplementary file 2 — Characteristics of participants stratified by dropout. (DOCX 19 kb) [file 12877_2018_860_MOESM2_ESM.docx]

**Additional file 2**: Characteristics of participants at t_1_ stratified by dropout

**Table A2**: Characteristics of participants at t_1_ stratified by dropout (n = 809)

|  |  | **N** | **No dropouts**  **(69.6%)** | **Dropouts**  **(30.4%)** | **P-value** |
| --- | --- | --- | --- | --- | --- |
| **Utilization of long-term care** | yes | 809 | 99 (17.6%) | 99 (40.2%) | **< 0.0001**^b^ |
| **Predisposing factors** |  |  |  |  |  |
| Age in years | total | 809 | 77.2 (6.0) | 81.2 (6.4) | **< 0.0001**^a^ |
| Sex | female | 809 | 273 (48.5) | 129 (52.4%) | 0.3014^b^ |
| Education | low | 809 | 359 (63.8%) | 188 (76.4%) | **0.0018**^b^ |
|  | middle |  | 118 (21.0%) | 35 (14.2%) |  |
|  | high |  | 86 (15.3%) | 23 (9.4%) |  |
| **Enabling factors** |  |  |  |  |  |
| Living arrangement | alone | 809 | 177 (31.4%) | 105 (42.7%) | **0.0020**^b^ |
| Per capita income in €/ month | total | 766 | 1173.8 (603.9) | 1056.0 (508.5) | **0.0059**^a^ |
|  | < 875 € |  | 121 (22.5%) | 60 (26.3%) | **0.0690**^b^ |
|  | 875-1,124 € |  | 132 (24.5%) | 66 (29.0%) |  |
|  | 1,125-1,374 € |  | 131 (24.4%) | 57 (25.0%) |  |
|  | ≥ 1,375 € |  | 154 (28.6%) | 45 (19.7%) |  |
| **Need factors** |  |  |  |  |  |
| Multimorbidity in no. of chronic conditions | total | 800 | 2.4 (1.5) | 3.0 (1.6) | **< 0.0001**^a^ |
| Disability score (HAQ-DI) | total | 808 | 0.368 (0.5) | 0.817 (0.9) | **< 0.0001**^a^ |

HAQ-DI: Health Assessment Questionnaire Disability Index

Bold numbers: significant at p ≤ 0.05

N = 809 due to one excluded individual at t_2_ because of missing information in utilization of long-term care (see subchapter “Statistical analysis”)

Data presented as n (%)/ mean (standard deviation) | any discrepancies in percentages due to rounding

^a^ based on t-test ^b^ based on chi2-test
