# Supplementary material for: Determinants for utilization and transitions of long-term care in adults 65+ in Germany: results from the longitudinal KORA-Age study
Source: BMC Geriatr. 2018 Jul 31;18:172. doi: 10.1186/s12877-018-0860-x (PMC6069853; doi:10.1186/s12877-018-0860-x)
Supplement: Supplementary file 3 — Time-varying characteristics of participants. (DOCX 18 kb) [file 12877_2018_860_MOESM3_ESM.docx]

**Additional file 3:** Time-varying characteristics of participants available at t_1_ and t_2_

**Table A3**: Time-varying characteristics of participants available at t_1_ and t_2_ (both n = 563)

|  |  | **t_1_** | **t_2_** |
| --- | --- | --- | --- |
| **Utilization of long-term care** | yes | 99 (17.6%) | 180 (32.0%) |
| **Predisposing factors** |  |  |  |
| Age in years | total | 77.2 (6.0) | 81.3 (6.0) |
| **Enabling factors** |  |  |  |
| Living arrangement | alone | 177 (31.4%) | 354 (62.9%) |
| **Need factors** |  |  |  |
| Disability score (HAQ-DI)^a^ | total | 0.368 (0.5) | 0.598 (0.8) |

HAQ-DI: Health Assessment Questionnaire Disability Index

Only time-varying variables are listed

Data presented as n (%)/ mean (standard deviation) | any discrepancies in percentages due to rounding

^a^ missings: n = 2 at t_1_, n = 1 at t_2_
